# Supplementary material for: Variation in HIV care and treatment outcomes by facility in South Africa, 2011–2015: A cohort study
Source: PLoS Med. 2021 Mar 31;18(3):e1003479. doi: 10.1371/journal.pmed.1003479 (PMC8012100; doi:10.1371/journal.pmed.1003479)
Supplement: S1 Fig — Figure shows how the sample of facilities used in the analysis was identified after applying exclusion criteria. (PDF) [file pmed.1003479.s002.pdf]

S1 Fig. Exclusion flowchart

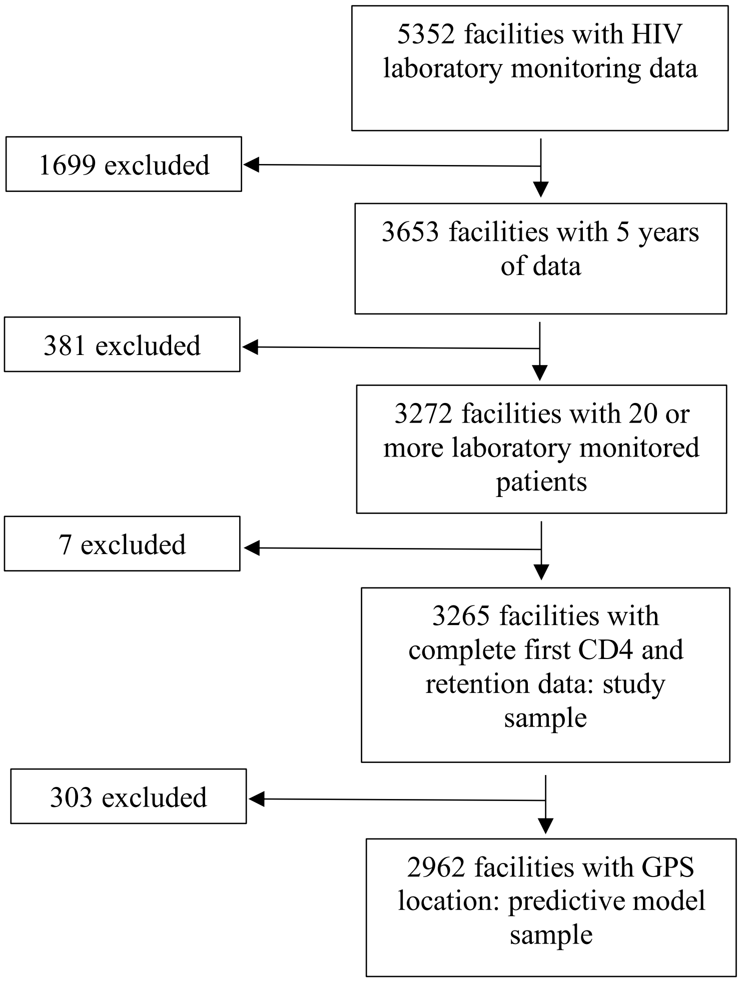

Supporting information for: **Bor J, Gage A, et al. Variation in HIV care and treatment outcomes by facility in South Africa, 2011-2015: a cohort study.** ***PLOS Medicine.***
