# Supplementary material for: Variation in HIV care and treatment outcomes by facility in South Africa, 2011–2015: A cohort study
Source: PLoS Med. 2021 Mar 31;18(3):e1003479. doi: 10.1371/journal.pmed.1003479 (PMC8012100; doi:10.1371/journal.pmed.1003479)
Supplement: S2 Fig — Figure displays heat maps showing the covariate-adjusted mean quality score and changes in the quality score over time. (PDF) [file pmed.1003479.s003.pdf]

S2 Fig. Geographic variation in adjusted quality score (left) and changes in quality over time (right), 2011-2015

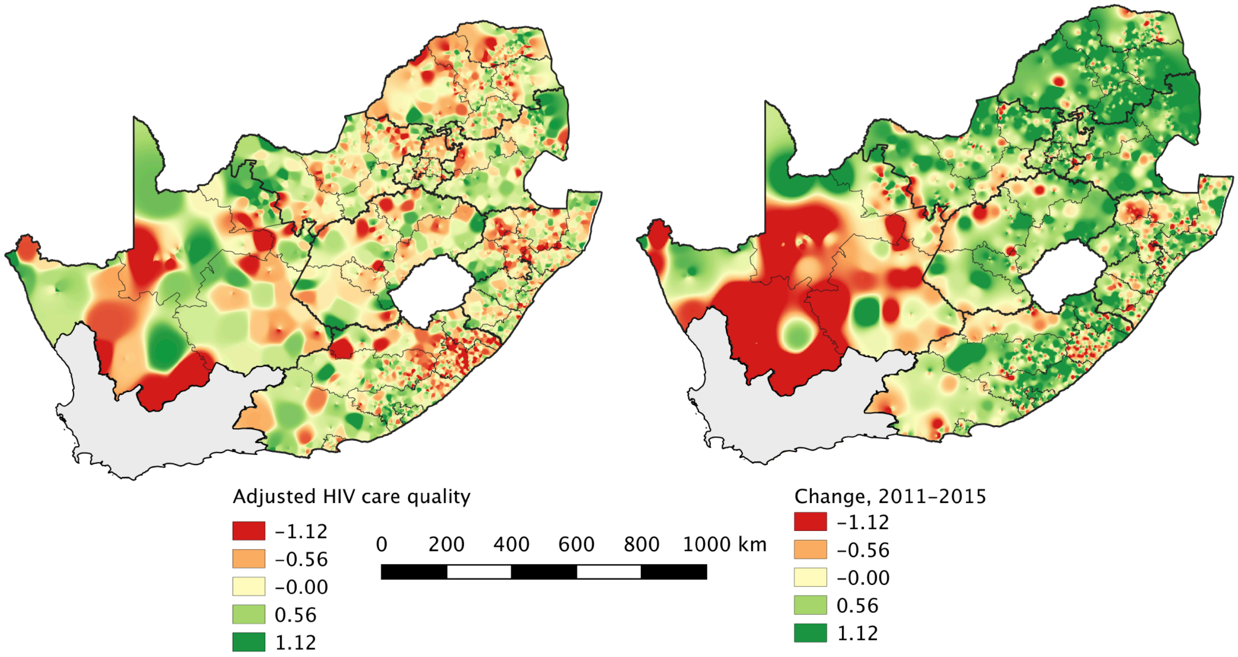

Note: Base map was obtained from the South African Municipal Demarcation Board (http://www.demarcation.org.za/).

Supporting information for: Bor J, Gage A, et al. Variation in HIV care and treatment outcomes by facility in South Africa, 2011-2015: a cohort study. *PLOS Medicine.*
