# Supplementary material for: Variation in HIV care and treatment outcomes by facility in South Africa, 2011–2015: A cohort study
Source: PLoS Med. 2021 Mar 31;18(3):e1003479. doi: 10.1371/journal.pmed.1003479 (PMC8012100; doi:10.1371/journal.pmed.1003479)
Supplement: S1 Table — Provided as a complement to Table 1, this Supporting information table shows means and standard deviations for the unbalanced panel including all facilities regardless of whether they had data for all years. (PDF) [file pmed.1003479.s004.pdf]

S1 Table. HIV quality indicators for all observed facilities[a]

Year		2011	2012	2013	2014	2015
Median first CD4 count	Mean	311.9	307.7	310.1	312.2	325.3
	SD	94.1	88.6	94.4	85.3	95.3
	N facilities	4,356	4,573	4,603	4,309	4,269
Retention 0-350	Mean	0.409	0.417	0.433	0.450	0.465
	SD	0.169	0.180	0.182	0.181	0.205
	N facilities	4,161	4,328	4,232	4,086	3,937
Retention 350+	Mean	0.354	0.349	0.354	0.370	0.399
	SD	0.174	0.172	0.162	0.164	0.194
	N facilities	4,161	4,333	4,255	4,118	3,995
Retention in care	Mean	0.774	0.763	0.770	0.765	0.719
	SD	0.129	0.132	0.132	0.127	0.172
	N facilities	4,281	4,522	4,552	4,282	4,235
Viral suppression	Mean	0.672	0.727	0.730	0.753	0.780
	SD	0.263	0.213	0.192	0.168	0.156
	N facilities	3,483	3,918	4,209	4,063	4,095
CD4 recovery	Mean	0.384	0.476	0.577	0.602	0.587
	SD	0.197	0.187	0.171	0.168	0.167
	N facilities	4,094	4,309	4,247	4,128	4,076
Viral monitoring after unsuppressed	Mean	0.229	0.242	0.272	0.311	0.373
	SD	0.221	0.205	0.205	0.195	0.198
	N facilities	3,171	3,678	3,991	3,924	3,940

[a]Excluding Western Cape province facilities. S1 Table replicates Table 1, but shows data for all facilities rather than the balanced panel 3265 facilities included in the analysis.

Supporting information for: Bor J, Gage A, et al. Variation in HIV care and treatment outcomes by facility in South Africa, 2011-2015: a cohort study. *PLOS Medicine.*
