# Supplementary material for: Variation in HIV care and treatment outcomes by facility in South Africa, 2011–2015: A cohort study
Source: PLoS Med. 2021 Mar 31;18(3):e1003479. doi: 10.1371/journal.pmed.1003479 (PMC8012100; doi:10.1371/journal.pmed.1003479)
Supplement: S2 Table — Provided as a complement to Table 2, this Supporting information table displays the correlation matrix between the underlying indicators and the summary quality measure for those facilities that are observed for 4 years (rather than 5 years), enabling inclusion of additional facilities. (PDF) [file pmed.1003479.s005.pdf]

S2 Table. HIV quality indicators using 4 year panel of 3,440 facilities, 2012-2015

	Median first CD4 count	Retention after first CD4 0-350	Retention after first CD4 350+	Retention starting 6 mo after CD4	Viral suppression	CD4 recovery	Factor 1
Median first CD4 count	1						-0.0942
Retention after first CD4 0-350	0.0150	1					0.6654
Retention after first CD4 350+	0.0615	0.4951	1				0.5532
Retention starting 6 mo after CD4	-0.0999	0.5057	0.4028	1			0.6549
Viral suppression	-0.1618	0.1878	0.0971	0.2110	1		0.3814
CD4 recovery	-0.2093	0.1192	0.0576	0.1631	0.3400	1	0.2850
Monitoring after unsuppressed	0.0119	0.2049	0.1572	0.2696	0.2445	0.0844	0.3618

Supporting information for: Bor J, Gage A, et al. Variation in HIV care and treatment outcomes by facility in South Africa, 2011-2015: a cohort study. *PLOS Medicine.*
