# Supplementary material for: Variation in HIV care and treatment outcomes by facility in South Africa, 2011–2015: A cohort study
Source: PLoS Med. 2021 Mar 31;18(3):e1003479. doi: 10.1371/journal.pmed.1003479 (PMC8012100; doi:10.1371/journal.pmed.1003479)
Supplement: S3 Table — Table displays factor loadings of the underlying indicators with the top 3 factors, the eigenvalues of those 3 factors, and the uniqueness of the indicators—i.e., the residual variance not explained by the factors. (PDF) [file pmed.1003479.s006.pdf]

S3 Table. Factor loadings of HIV care quality in 3253 facilities

Variable	Factor1	Factor2	Factor 3	Uniqueness
Median first CD4 count	-0.046	-0.341	0.138	0.863
Retention after first CD4 0-350	0.675	-0.187	-0.031	0.509
Retention after first CD4 350+	0.580	-0.257	-0.018	0.597
Retention starting 6 mo after CD4	0.637	-0.098	-0.041	0.583
Viral suppression	0.343	0.437	0.048	0.689
CD4 recovery	0.269	0.456	-0.018	0.719
Monitoring after unsuppressed	0.357	0.138	0.145	0.833
Eigenvalue	1.517	0.644	0.045	

Supporting information for: Bor J, Gage A, et al. Variation in HIV care and treatment outcomes by facility in South Africa, 2011-2015: a cohort study. *PLOS Medicine.*
