# Supplementary material for: Variation in HIV care and treatment outcomes by facility in South Africa, 2011–2015: A cohort study
Source: PLoS Med. 2021 Mar 31;18(3):e1003479. doi: 10.1371/journal.pmed.1003479 (PMC8012100; doi:10.1371/journal.pmed.1003479)
Supplement: S4 Table — Table displays descriptive statistics for the municipalities where the facilities in the analysis were located. (PDF) [file pmed.1003479.s007.pdf]

S4 Table. Characteristics of 207 municipalities

Variable	Mean	Std. Dev.	Min	Max
% households in poverty	0.376	0.106	0.171	0.620
% households with piped water	0.642	0.274	0.063	0.981
% households with electricity	0.789	0.153	0.145	0.969
% population over 60	0.086	0.020	0.037	0.164
% population literate	0.781	0.072	0.582	0.954
% households moved	0.119	0.063	0.015	0.423
% households with internet	0.255	0.075	0.105	0.513
% majority black households	0.820	0.241	0.028	1.000

Supporting information for: Bor J, Gage A, et al. Variation in HIV care and treatment outcomes by facility in South Africa, 2011-2015: a cohort study. *PLOS Medicine.*
