# Supplementary material for: Variation in HIV care and treatment outcomes by facility in South Africa, 2011–2015: A cohort study
Source: PLoS Med. 2021 Mar 31;18(3):e1003479. doi: 10.1371/journal.pmed.1003479 (PMC8012100; doi:10.1371/journal.pmed.1003479)
Supplement: S5 Table — Table displays an expanded predictive model as a supplement to Table 3. Some facilities were not included in the predictive model due to missing data on predictors. (PDF) [file pmed.1003479.s008.pdf]

S5 Table. Full model predictors of HIV care quality, 2962 facilities

	Bivariate models		Multivariable model	
	Beta	95% CI	Beta	95% CI
Facility characteristics				
log N patients	0.14	(0.11,0.16)	0.12	(0.10,0.14)
Facility type (clinic ref.)				
District or CHC	-0.83	(-0.92,-0.75)	-0.93	(-1.01,-0.84)
Provincial or national hospital	-1.08	(-1.20,-0.96)	-1.13	(-1.25,-1.01)
Municipality characteristics				
Rural	0.15	(0.07,0.24)	0.14	(0.07,0.22)
% households in poverty	0.02	(-0.04,0.09)	-0.09	(-0.21,0.03)
% majority black households	0.02	(-0.03,0.07)	-0.01	(-0.09,0.06)
% population over 60	0.04	(-0.03,0.10)	0.15	(0.09,0.22)
% population literate	0.02	(-0.06,0.10)	-0.05	(-0.15,0.05)
% households moved	-0.05	(-0.11,0.01)	-0.04	(-0.12,0.04)
% households with internet	0.07	(-0.01,0.15)	0.03	(-0.09,0.15)
% households with piped water	-0.02	(-0.09,0.04)	0.05	(-0.06,0.16)
% households with electricity	-0.06	(-0.13,0.00)	0.05	(-0.02,0.13)
Province (Eastern Cape ref)				
Free State	0.26	(-0.10,0.53)	0.07	(-0.16,0.31)
Gauteng	0.26	(-0.07,0.59)	0.20	(-0.09,0.50)
KwaZulu-Natal	0.64	(0.45,0.83)	0.64	(0.47,0.82)
Limpopo	-0.13	(-0.34,0.09)	-0.21	(-0.40,-0.02)
Mpumalanga	-0.21	(-0.44,0.02)	-0.19	(-0.39,0.01)
North West	0.01	(-0.24,0.26)	-0.13	(-0.34,0.08)
Northern Cape	-0.08	(-0.35,0.20)	-0.24	(-0.50,0.03)
Year (2011 ref)				
2012	0.11	(0.08,0.14)	0.09	(0.06,0.12)
2013	0.33	(0.30,0.36)	0.30	(0.27,0.33)
2014	0.48	(0.45,0.52)	0.44	(0.40,0.47)
2015	0.55	(0.51,0.59)	0.49	(0.46,0.53)
Constant			-1.03	(-1.21,-0.84)

Supporting information for: Bor J, Gage A, et al. Variation in HIV care and treatment outcomes by facility in South Africa, 2011-2015: a cohort study. *PLOS Medicine.*
