# Supplementary material for: Variation in HIV care and treatment outcomes by facility in South Africa, 2011–2015: A cohort study
Source: PLoS Med. 2021 Mar 31;18(3):e1003479. doi: 10.1371/journal.pmed.1003479 (PMC8012100; doi:10.1371/journal.pmed.1003479)
Supplement: S6 Table — Table displays an alternate predictive model estimated for facilities that are observed for 4 years, enabling inclusion of additional facilities. Some facilities were not included in the predictive model due to missing data on predictors. (PDF) [file pmed.1003479.s009.pdf]

S6 Table. Predictors of HIV quality using 4 year panel of 3,116 facilities

	Coef.	[95% CI]
Facility characteristics		
log N patients	0.12	(0.10,0.14)
Facility type (clinic ref.)		
District or CHC	-1.00	(-1.09,-0.91)
Provincial or national hospital	-1.13	(-1.25,-1.00)
Municipality characteristics		
Rural	0.11	(0.04,0.19)
% households in poverty	-0.10	(-0.17,-0.02)
% majority black households	-0.01	(-0.08,0.07)
% population over 60	0.16	(0.10,0.21)
Province (Eastern Cape ref)		
Free State	0.23	(0.01,0.44)
Gauteng	0.25	(-0.03,0.53)
KwaZulu-Natal	0.56	(0.39,0.73)
Limpopo	-0.06	(-0.25,0.12)
Mpumalanga	-0.03	(-0.23,0.17)
North West	-0.08	(-0.28,0.11)
Northern Cape	-0.25	(-0.51,0.01)
Year (2012 ref)		
2013	0.21	(0.18,0.23)
2014	0.35	(0.32,0.38)
2015	0.38	(0.35,0.42)
Constant	-1.00	(-1.19,-0.82)

Supporting information for: Bor J, Gage A, et al. Variation in HIV care and treatment outcomes by facility in South Africa, 2011-2015: a cohort study. *PLOS Medicine.*
