# Supplementary material for: Variation in HIV care and treatment outcomes by facility in South Africa, 2011–2015: A cohort study
Source: PLoS Med. 2021 Mar 31;18(3):e1003479. doi: 10.1371/journal.pmed.1003479 (PMC8012100; doi:10.1371/journal.pmed.1003479)
Supplement: Attachment — Submitted filename: bor.pdf [file pmed.1003479.s010.pdf]

This paper uses routine data to analyse quality of care in HIV in South Africa.

I was asked for a statistical report and I interpret that to include all aspects of the design and conduct of the study.

Points of detail

Page 4 Although as Supplementary Figure 1 will later reveal many facilities have been excluded as a result these decisions all seem reasonable to me.

Page 5 I can understand the problem of people who move facilities but using first facility throughout seems hard to justify.

Page 7 I would have used proportion rather than share here and it made me hesitate for a moment when first reading.

Page 8 Selecting a subset of variables in a way driven by the data leads to a model which is unlikely to replicate (Babyak, 2004). In fact looking later at the small number of variables which are dropped in Table 3 I would suggest using a full model would be superior on all grounds. Some countries have defined a measure of area–level deprivation which would replace the various municipality variables but I assume such a thing is not available to the authors as it would have simplified the model.

Page 8 Strictly speaking multivariable is meant here (Hidalgo and Goodman, 2013). Multivariate means multiple variables on the left hand side. The authors could in principle have fitted a multivariate model but that is not in fact what they did.

Page 10 I did not grasp what the sentence starting 'Perhaps the most important' meant. Does this mean that facility performance is stable over time? Perhaps re–write?

Page 18 Typo, Loneway

Page 18, Figure 2 I do not think the use of different saturations works well when there is so much overprinting. Five separate plots might be better. Given the message the authors seem to want us to draw from the plot I wonder whether we need to see the points at all and whether a single plot showing the convex hull of each of the five groups would be more revealing.

Table 3 Some of the changes in $\hat{\beta}$ between the bivariate models and the multivariable model seem very striking. For instance moving from -0.41 to 0.31 is a reversal and moving from 2.01 to 8.91 is unusually extreme. There must be something about the inter-relationship of the predictors which accounts for this. I think more explanation is needed.

Table 3 I do not understand either of the table footnotes here I am afraid.

Points of more substance

There is quite a bit I do not understand about the use of factor analysis here.

As I understand it the input to the process would have been a correlation matrix. Since this is 7×7 I would have thought the eigenvalues should have summed to 7 but given the first three as shown in Table S2 that cannot be the case.

Very little of the variance is explained by the first factor (or indeed all three) judging from the commonalities given in Table S2. Many people would use a cut–off of 0.3 or even 0.4 for saying a variable contributes to a factor which makes the first factor shown here reliant on only a few manifest variables. Table 3 confirms that the inter–correlations are mostly quite weak.

Although these are all plausible measures of quality of care I am not convinced the authors have demonstrated that in fact they do measure a single latent variable.

Summary

Quite a bit of clarification asked for and a point about the latent variable to address.

Michael Dewey

References

M A Babyak. What you see may not be what you get: a brief, nontechnical introduction to overfitting in regression–type models. *Psychosomatic Medicine*, 66:411–421, 2004.

B Hidalgo and M Goodman. Multivariate or multivariable regression. *American Journal of Public Health*, 103:39–40, 2013.
