# Supplementary material for: Variation in HIV care and treatment outcomes by facility in South Africa, 2011–2015: A cohort study
Source: PLoS Med. 2021 Mar 31;18(3):e1003479. doi: 10.1371/journal.pmed.1003479 (PMC8012100; doi:10.1371/journal.pmed.1003479)
Supplement: Attachment — Submitted filename: Quality, RR1 Response Letter_14oct2020.docx [file pmed.1003479.s011.docx]

**RESPONSES TO EDITOR AND REVIEWER COMMENTS**

*****Requests from the editors:**

*1. Please finalize the data statement, adding details for a non-author contact.*

🡪 We have added the following statement: “Access to primary data is subject to restrictions owing to privacy and ethics policies set by the South African Government. Requests for access to the data can be made to the National Health Laboratory Services directly (<http://www.nhls.ac.za/>) and require a full protocol submission. Inquiries can be made to Babatyi Malope-Kgokong, National Manager: Academic Affairs and Research at NHLS at [Babatyi.kgokong@nhls.ac.za](mailto:Babatyi.kgokong@nhls.ac.za).”

*2. Please adapt the title to better match journal style. We suggest "Variation in HIV care and treatment outcomes by facility in South Africa, 2011-2015: a cohort study".*

🡪 We have revised the title accordingly.

*3. Please make the abstract subheadings "Background/Methods and findings/Conclusions".*

🡪 We have revised the subheadings accordingly.

*4. Please add a new final sentence to the "Methods and findings" subsection, summarizing 2-3 of the study's main limitations.*

🡪 We have included an additional sentence in the abstract: “The study’s limitations include an inability to fully adjust for underlying patient risk and reliance on laboratory data which does not capture all relevant domains of quality.”

*5. Please begin the "Conclusions" subsection with "In this study, we observed that ..." or similar.*

🡪 We have revised the abstract accordingly.

*6. After the abstract, please add a new and accessible "author summary" section in non-identical prose. You may find it helpful to consult one or two recent research papers published in PLOS Medicine to get a sense of the preferred style.*

🡪 We have added in an author summary section after the abstract.

*7. Please adapt the final sentence of your Introduction to remove elements of discussion (e.g., "... complements ongoing efforts ..."), which can be moved to the discussion section if you wish.*

🡪 We have moved these sentences to the discussion section.

*8. At the start of the methods section, should that be "2011-2015"? Similarly, early in the Discussion section (currently "2011-2017").*

🡪 We have corrected the Discussion section to indicate that the quality scores are from 2011-2015. The laboratory data is for the period 2011-2016, though because many of the quality measures have a 6 or 12 month follow up period that are assigned to the beginning of the interval, the quality measures are only defined from 2011-2015. We have clarified this distinction in the methods section: “With patient laboratory data from January 2011 through December 2016, we were able to define annual facility quality measures for 2011-2014 and for the first six months of 2015.”

*9. Early in the methods section of your main text, please state whether or not the study had a protocol or prespecified analysis plan, and if so attach the relevant document as a supplementary file (referred to in the text). Please highlight analyses that were not prespecified.*

🡪 This study did not have a protocol or pre-specified analysis plan, which we have added to the methods section

*10. Please add a sentence on ethics approval to the methods section - e.g., noting that this was not required.*

We have added: “**Ethics approval.** The study was approved by the Human Research Ethics Committee of the University of the Witwatersrand and the Boston University Institutional Review Board for use of de-identified data with a waiver of consent.”

*11. Please restructure the early part of the discussion section of your main text, so that the first paragraph provides a summary of the study's findings.*

🡪 We have rearranged the discussion section to make the first paragraph of a summary of the key findings.

*12. Throughout the text, please move reference call-outs to fall before punctuation (e.g., "... measures of quality [9,21].".*

🡪 We have revised the formatting accordingly.

*13. Please spell out the institutional author name for reference 24, and for other references where necessary.*

🡪 We have reviewed all institutional references and updated them, spelling out the full institution name in the author position.

*14. Please add a completed checklist for the most appropriate reporting guideline, e.g., STROBE or RECORD, referred to in the methods section of your main text. In the checklist, please refer to individual items by section (e.g., "Methods") and paragraph number rather than by line or page numbers, as the latter generally change in the event of publication.*

Completed.

*****Comments from academic editor:**

*I have now read this manuscript, which I find interesting and contributing to our knowledge of HIV programmes. The methods applied are novel, and methodologically sound. The focus of assessing quality of care provided at facilities is of interest to programme makers, and the combination tool developed to assess that quality is new - allowing for patient-outcomes as per call from the 2018 Lancet Global Health Commission of High Quality Systems. They have used the South African NHLS database, in combination with the 2011 census data on municipality characteristics. So far so good.*

*1. Limitations includes those mentioned by the authors, but also the nature of the data - they have data from the NHLS from Jan 2011 to Dec 2016, but are able to use data from 2011 to mid 2015 only. They do not discuss the relevance of their findings to the current programmes (with more test and immediate treatment, less reliance on CD4 and only annual VL). Further, they used the 2011 census data - which is the last census available (and appropriate for the 2011-2015 data they use), but should mention whether there could be subsequent changes at municipality level that could be important. They also do not discuss how they allow for previous patient-level and facility factors for people who move their care from one to another facility (eg can they see whether people move from lower care quality to higher care quality).*

🡪 Thank you for these comments. The analysis does use data from 2016, but due to the look-back period required of the retention measures, the quality measures are only defined through 2015. We have attempted to clarify this in the methods section.

We have added the following text to the discussion section to make the relevance to today’s programming more clear: “Finally, South Africa – and other countries – have made significant changes to their treatment programs in recent years, with the advent of universal test-and-treat which may affect measurement of quality-of-care. After September 2016, national guidelines specify that CD4 counts should still be taken at clinical presentation to assess for potential treatment complications and to target additional care to patients with advanced disease. However, because CD4 counts are no longer used to determine treatment eligibility, facilities may be less rigorous in collecting these laboratory results. Additionally, CD4 counts are no longer collected after initiation of treatment, preventing assessment of CD4 recovery. As models of care shift, the quality score will have to be updated to accommodate these changes.”

We included these limitations, “We also only use laboratory data through December 2016 before universal test-and-treat was implemented and do not yet have enough data post implementation for comparison. Fourth, there may be changes in the municipality characteristics over the study period that are not captured in the 2011 census. Use of the municipality data also assumes that the population use a facility in their community and don’t travel long distances for care.”

*2. Their finding that the median CD4 count at presentation was not identified as a factor in the quality of care model may be because there was not much heterogeneity between facilities in that count? Also, with the current guidelines what is the relevance of CD4 count in quality assessment?*

🡪 In response to this comment, we went back to the data to assess heterogeneity in median CD4 count by facility. We find that there is actually quite substantial variation in median CD4 count. For example, in 2011 when the facility average median first CD4 was 303, there was a standard deviation of 57 and IQR of 74. These data are shown in Table 1. Variation did decrease slightly (SD = 47 in 2015) over the period of the study as the worst performing facilities improved, but this trend was not dissimilar to the other assessed quality measures. We have now revised the first paragraph in the results section to highlight the standard deviations of the underlying measures, in order to emphasize the substantial heterogeneity across facilities:

Results, p9-10: “Our analysis included data on 3265 facilities with observations for each year, 2011-2015. **Table 1** presents the unweighted mean and standard deviation (SD) for each of the facility performance measures for each year. Median CD4 count at presentation increased from a mean of 303 (SD = 57) cells/mm^3^ in 2011 to a mean of 312 (SD = 47) cells/mm^3^ in 2015, as the worst performing facilities improved. From 2011 to 2015, the average share of patients retained 12 months after presentation increased from 42% (SD = 13%) to 47% (SD = 19%) for patients with a first CD4 count < 350, and from 36% (SD = 14%) to 41% (SD = 17%) for patients with a first CD4 count ≥ 350. Prevalent retention among those already in care fell modestly, from 78% (SD = %9) to 73% (SD = 13%), though there was less variation across facilities than in other measures. Viral suppression increased from 66% (SD = 26%) to 79% (SD = 12%), and CD4 recovery from 39% (SD = 17%) to 60% (SD = 14%). The average share of patients monitored after an unsuppressed viral load increased from 23% (SD = 22%) to 38% (SD = 18%) over the period of study. **S1 Table** shows means and standard deviations for the unbalanced panel including all facilities regardless of whether they had data for all years. In summary, the data reveal substantial variation in the underlying measures across facilities, as well as large increases in viral suppression, CD4 recovery, and monitoring after potential viral failure over time.”

🡪 We have also revised the Discussion section as follows: “After September 2016, national guidelines specify that CD4 counts should still be taken at clinical presentation to assess for potential treatment complications and to target additional care to patients with advanced disease. However, because CD4 counts are no longer used to determine treatment eligibility, facilities may be less rigorous in collecting these laboratory results. Additionally, CD4 counts are no longer collected after initiation of treatment, preventing assessment of CD4 recovery. As models of care shift, the quality score will have to be updated to accommodate these changes.”

*3. And they should also discuss in more detail than they do currently, what improvements could be made at quality level to improve the overall quality score?*

🡪 We have added to the discussion: “Facilities could improve their quality by improving the any of these underlying components. For example, in the highest quintile of facilities, 82% of patients achieved viral suppression; whereas 60% were suppressed in the lowest quintile. If all facilities achieved viral suppression at levels equal to facilities in the highest quintile of quality, overall suppression would be increased by 22 percentage points in the lowest quintile and by 3.5 percentage points nationally – from 81.5% to 85% suppressed.”

*****Comments from the reviewers:**

***** Reviewer #1:**

This paper uses routine data to analyse quality of care in HIV in South Africa. I was asked for a statistical report and I interpret that to include all aspects of the design and conduct of the study.

**A. Points of detail**

*1) Page 4 Although as Supplementary Figure 1 will later reveal many facilities have been excluded as a result these decisions all seem reasonable to me.*

We have included a reference to the Supplementary figure in the text when discussing the exclusion criteria.

*2) Page 5 I can understand the problem of people who move facilities but using first facility throughout seems hard to justify.*

We clarify that we define retention episodes based on the facility where that current episode began. (A patient may have had prior care episodes at other facilities before the current episode.) We believe that assigning episodes to the facility where they began is a defensible choice. The reason is that a retention episode can end in either retention or attrition. However, given the typical time between laboratory tests, “transfer”, which requires at least 2 tests, implies retention almost by definition. As a result, if we assigned transfers to the receiving clinic, then the receiving clinic gets a large number of “retained” cases without commensurate “attrition cases”; and the sending clinic has all the “attrition” cases and gets no credit for patients who were retained in care but went elsewhere. As a result, this will unfairly bias downwards retention in care for those facilities that are common entry-points for HIV care.

We explain in the Methods section, p6:

“In cases of transfer, care episodes were assigned to the facility where the episode began, i.e. the sending facility, to avoid bias in retention estimates. Because unsuccessful transfers, i.e. transfers that do not lead to retention in care, are not identified in the data and cannot be linked to a receiving facility, assigning successful transfers to receiving facilities would have led to upward bias in retention at those facilities and downward bias at sending facilities.”

*3) Page 7 I would have used proportion rather than share here and it made me hesitate for a moment when first reading.*

We have revised the text accordingly.

*4) Page 8 Selecting a subset of variables in a way driven by the data leads to a model which is unlikely to replicate (Babyak, 2004). In fact looking later at the small number of variables which are dropped in Table 3 I would suggest using a full model would be superior on all grounds.*

Thank you for this suggestion. We considered this reviewer comment alongside the reviewer’s other feedback that multicollinearity among predictors could obscure important relationships. After reviewing all the municipal-level predictors, we decided to narrow our list to a smaller set of predictors (poverty, black, elderly and rural) that measure conceptually distinct theoretical constructs and exhibit less multicollinearity. We have revised Table 3 to include just these covariates at the municipal level. At the same time, to address this reviewer comment, we have included an appendix (S7) that includes all of the previously identified municipality variables and presents both bivariate regressions as well as the full multivariable model for all of these predictors. We hope that this transparent approach addresses both reviewer concerns.

*5) Some countries have defined a measure of area–level deprivation which would replace the various municipality variables but I assume such a thing is not available to the authors as it would have simplified the model.*

Thank you for this suggestion. While there are proposed measures of area-level deprivation in South Africa, there is not a single standardized measure that is widely accepted and used. As indicated above, we have narrowed down the list of predictors in the main model to look at differences between sub-groups.

*6) Page 8 Strictly speaking multivariable is meant here (Hidalgo and Goodman, 2013). Multivariate means multiple variables on the left hand side. The authors could in principle have fitted a multivariate model but that is not in fact what they did.*

We have corrected the text accordingly.

*7) Page 10 I did not grasp what the sentence starting ‘Perhaps the most important’ meant. Does this mean that facility performance is stable over time? Perhaps re–write?*

We have removed this sentence in cutting down the text.

*8) Page 18 Typo, Loneway*

This is the name of a Stata command. We have clarified: “Reliability within facility was 0.842, computed using Stata’s loneway command, which fits one-way analysis-of-variance (ANOVA) models.”

*9) Page 18, Figure 2 I do not think the use of different saturations works well when there is so much overprinting. Five separate plots might be better. Given the message the authors seem to want us to draw from the plot I wonder whether we need to see the points at all and whether a single plot showing the convex hull of each of the five groups would be more revealing.*

We have reformatted this graph to show ellipses of the distribution of the data by tertile of facility size, which helps to clarify the message that there is a tighter correlation among larger facilities.

*10) Table 3 Some of the changes in βˆ between the bivariate models and the multivariable model seem very striking. For instance moving from −0.41 to 0.31 is a reversal and moving from 2.01 to 8.91 is unusually extreme. There must be something about the inter-relationship of the predictors which accounts for this. I think more explanation is needed.*

Please see our response to (4), above. In the revised model presented in Table 3, there is no longer such a large swing in the coefficients between the bivariate and multivariable model. The full model in S7 still has some substantial differences between the bivariate and multivariable models among the municipality characteristics, but this is not unexpected given multicollinearity among some of these predictors.

Based on the revised results, we have added the following text to the results section:

“Poorer municipalities had lower quality of care, with a one-standard deviation increase in the poverty rate associated with a -0.10 standard deviation reduction in quality of care. Conditional on area poverty and other predictors, percent Black African was not significantly associated with lower quality. **S7 Table** displays associations with additional population predictors.”

*11) Table 3 I do not understand either of the table footnotes here I am afraid.*

In revising Table 3, we no longer have the prior footnotes.

**B. Points of more substance**

*There is quite a bit I do not understand about the use of factor analysis here. As I understand it the input to the process would have been a correlation matrix. Since this is 7 × 7 I would have thought the eigenvalues should have summed to 7 but given the first three as shown in Table S2 that cannot be the case. Very little of the variance is explained by the first factor (or indeed all three) judging from the commonalities given in Table S2. Many people would use a cut–off of 0.3 or even 0.4 for saying a variable contributes to a factor which makes the first factor shown here reliant on only a few manifest variables. Table 3 confirms that the inter–correlations are mostly quite weak. Although these are all plausible measures of quality of care I am not convinced the authors have demonstrated that in fact they do measure a single latent variable.*

We chose to use Factor Analysis because we sought to assess whether persistent, underlying characteristics of facilities – which are mostly unobserved, but could include facility staff attitudes, management expertise, organizational culture, routine procedures of care, as well as patient populations served – explain variation in outcome-based measures of the care cascade. We label this summary of factors associated with good outcomes as “quality of care”.

The reviewer is correct: the input is a correlation matrix, which is shown in Table 2.

The reviewer writes: *“Since this is 7 × 7 I would have thought the eigenvalues should have summed to 7 but given the first three as shown in Table S2 that cannot be the case.”* The sum of eigenvalues is equal to the number of variables in principle components analysis, but not necessarily in factor analysis. Whereas PCA chooses factors that minimize the total unexplained variance – and therefore choose factors that explain all the variance; factor analysis chooses factors that predict the structure of the correlation matrix while allowing for unexplained variation. The eigenvalues would sum to 7 under the assumption that there is no uniqueness – i.e. that there is no residual unexplained variability in the variables. (This would mimic the assumption of PCA.) However, we find that there is substantial uniqueness – likely due to measurement error, as we explain below. Thus, the sum of the eigenvalues being less than 7 reflects that there is residual variance not explained by the factors.

The reviewer writes: *“Very little of the variance is explained by the first factor (or indeed all three) judging from the commonalities given in Table S2. Many people would use a cut–off of 0.3 or even 0.4 for saying a variable contributes to a factor which makes the first factor shown here reliant on only a few manifest variables. Table 3 confirms that the inter–correlations are mostly quite weak.”* The variance not explained is given by “uniqueness” in Table S2, and ranges from 50% to 86%. It is important to stress that the variance not explained includes substantial measurement error (noise) resulting from both small samples (sampling error) and randomness in the underlying data. Therefore, we would not expect a particularly high “variance explained”. Similarly, we would not expect very high correlations between the variables (Table 3), nor would we expect very high correlations of each variable with the underlying factor (Table 2). If we used a cut-off of 0.3, we would have excluded a measure – CD4 recovery – with a correlation of 0.26. As we did not set a threshold a priori, exclusion of this measure would be arbitrary. We could have also excluded median CD4 at presentation – which was found, *ex post*, NOT to be correlated with the other variables. But since the correlation of this measure with the quality score was effectively zero, it had very little impact on the estimated quality score.

The reviewer writes: “*Although these are all plausible measures of quality of care I am not convinced the authors have demonstrated that in fact they do measure a single latent variable.”* Given the extent of measurement error in the underlying constructs, the signal:noise ratio is low. Nevertheless, by leveraging the covariance of the underlying constructs, we extracted a latent factor with higher signal:noise. The measure had high persistence year to year, and the average score had a reliability of 0.84, indicating that our approach was useful in distinguishing between high- and low-scoring facilities. Our interpretation of the first factor as “quality-of-care” is based on our theory-driven selection of the underlying metrics. Further, the second factor had less than half the variance explained relative to the first factor and the underlying variables did not correlate with the second factor in a way predicted by theory. The third factor had a very low association with the underlying predictors, and the higher factors were negative (which is possible in factor analysis, although not in PCA).

We have added some explanation of the above to the text in the results section: “We note substantial residual variation in the component measures not explained by the factors, as evidenced by high “uniqueness” values in **S2 Table**. However, it is important to stress that this unexplained variation includes substantial random noise due to small facility populations and does not therefore imply that the first factor was unimportant. By leveraging the covariance of different facility indicators, we are able to extract a facility level signal from the underlying data. Factor loadings on the first factor revealed moderate-to-high positive correlations with the three retention measures and low-to-moderate positive correlations with viral suppression, CD4 recovery, and viral monitoring if unsuppressed (**Table 2**). The first factor was not correlated with median first CD4 count, suggesting our quality measure captures aspects of care for patients already in care, but does not capture the extent to which facilities are able to screen patients for HIV earlier in infection. In contrast to the first factor, the second factor did not correlate with the underlying variables in a way predicted by theory. Therefore, we interpret only the first factor as our measure of quality of care.”

***** Reviewer #2:**

Bor et al. use a large national database with laboratory parameters to design a quality assessment methodology for facilities in South Africa. Looking at the huge number of data, complicated by missing values, changing guidelines and varying settings, the authors should be complicated for their effort, especially since the appear to have found an underlying measure of quality of HIV care that correlate with all cascade steps, except CD4 at presentation.

*1) I do not understand why they picked CD4 at presentation as a quality measure: it doesn't really say anything on how facilities perform with respect to testing; it is more a marker of the population, in particular how they might come forward to medical facilities - it is certainly not an actionable parameter: you cannot invite people to visit to your facility earlier. All other measures are issues where activities of the facilities can really improve the outcomes. The authors should clarify why they chose this in the first place; I am not surprised it didn't turn out as a significant step in their quality measure - I miss in the discussion if they want to keep it in their evaluation. In the same line: why is CD4 recovery a measure? This is very individually determined, and hardly a factor that a facility can improve (except by viral suppression, which is already a parameter)*

🡪 We sought to be ecumenical in our approach, including a wide range of measures that could reflect quality of care, with the constraint that all measures could use laboratory data only. In the end, the data showed that median CD4 at presentation was not correlated with the other variables, likely for the very reasons the reviewer describes. However, we stand by our decision to include it *ex ante*. Public health facilities are responsible for reducing HIV burden of disease through provision of HIV care and treatment – including HIV treatment-as-prevention – for a population catchment area. Facilities providing the *highest* quality of care should conduct active case-finding and testing outreach efforts in the community and also should partner with other HIV testing sites (e.g. occupational sites, mobile testing, etc), and ensure high rates of linkage to care for people who test positive. Such efforts would lead to identifying patients earlier in disease progression and to higher median CD4 counts at presentation.

We have now clarified why we include CD4 counts at presentation in the methods section: “A high median first CD4 count would therefore be a marker for high quality because the facility would be able to detect cases early through outreach and testing.”

*2) The introduction is long and for me it is not entirely clear, why they want to express quality in one score fabricated with a quite complicated statistical approach, where the outcome needs a lot of explanation. The charm of the cascade of care, is that one can see which step is the weakest part of the cascade. Furthermore, if one strives to get one score, why not the product of the several steps: for instance 70%retention x 80%cART x 80%suppression would make 0,45*

🡪 This is a great question. We believe our approach has several strengths vis-à-vis the cascade approach. First, the 90-90-90 HIV care cascade is a population parameter: the denominator for the first 90 is the underlying population of people living with HIV. It cannot be estimated at a facility level without facility catchment area prevalence estimates. Secondly, the care cascade, as it is typically used, reflects cross-sectional measures: is a patient on ART? Are they virally suppressed if on ART? However, our approach incorporates longitudinal measures that better capture quality of chronic disease management: is a patient still in care X months later? Third, by pooling information from several different measures, we reduce the influence of noise vis-à-vis the underlying quality signal.

We have added to the Discussion: “This measure has advantages over the common 90-90-90 cascade for measuring facility level quality: it can be measured without facility catchment area prevalence estimates and incorporates longitudinal rather than cross-sectional estimates that better reflect structure of chronic disease management.”

*3) The authors used the data from over 4000 South African facilities, but showed in their S1 Figure that 2300 facilities were excluded for various reasons. Although I understand their choice, I think they should have discussed in their limitations section what this means for their conclusions, especially since they conclude that larger centers appear to have a higher quality. The data of how many patients were not analyzed this way? How can quality be judged in the facilities that were excluded? Or will the authors now advise that hiv care can better be concentrated in larger facilities?*

We have added to the Discussion: “Third, as we have excluded very small facilities and facilities in the Western Cape, our findings not generalizable to these facilities. We found that large facilities performed better than small facilities, however this may not apply to very small facilities and other methods may be needed to account for the very large heterogeneity seen in those facilities.”

*4) I miss what the quality score tells an individual facility, since I presume all facilities will get their own score, so they can compare their performance longitudinally, but also with best practices. I expect that the score will be broken down to the individual steps of the cascade, so every facility can better understand which steps need more attention for improvement.*

We have revised the Discussion: “Sharing information with facilities on overall quality; on the component measures; and on changes over time may be an effective starting point for quality improvement.”

*5) Finally, I think overall the texts are too long and therefore not always easy to read. I would advise to shorten it, with more focus on the reason for choosing this approach, what individual facilities can do with "one individual score" and how this score is different from the already used cascade of care approach*

In revision, we have streamlined the presentation. We believe it reads more clearly but welcome further Editorial feedback. We have also added a discussion of the relevance of this score alongside more traditional cascade measures to the Discussion section, as described above.

***** Reviewer #3:**

*In this paper, the research team uses laboratory data (CD4+ count, viral load) from the NHLS in South Africa to construct a cohort of HIV+ individuals in eight of nine provinces, then combines a number of quality metrics derived from the lab data into a single quality measure. They then analyse the variation in this quality measure across space and time, and explore population-level and facility-level factors associated with quality. This is really interesting and thought-provoking, and builds on previous work from this group using this constructed lab cohort to provide insight into the national HIV programme. I found some of the main results quite surprising, particularly the apparent increase in quality over time (over a period when services and facilities were becoming very stretched). Whilst there are obvious limitations to analysing laboratory data without linked clinical data, I think these findings do provide some helpful insight and show the potential of the use of data to make inferences about quality of services and to guide decision making.*

*Minor comments
1.      I would suggest that the exclusion of Western Cape should be mentioned in the abstract so that it's clear this analysis is only based on data from eight provinces*

🡪 We have done so.

*2.      I would like to see some explanation for why the data for this analysis stopped in 2015/2016, especially if making an argument that this is something that could be potentially useful in real time to track service quality. Is the work on the lab cohort not ongoing?*

🡪 Work on the lab cohort is ongoing, and this paper provides a proof of concept. Because UTT changed some of the underlying measures – and may suggest new ones – we though it wise to stop at UTT implementation to avoid distracting from the main point of the paper. We do not currently have access to a long enough period post-UTT to separately identify quality and assess reliability of the measure over several years. We plan to update our approach for the UTT era in a future analysis.

We have added this limitation to the discussion section: “We also only use laboratory data through December 2016 before universal test-and-treat was implemented and do not yet have enough data post-implementation for comparison.”

*3.      Methods, p5 (Retention 12-months after first CD4 count <350) - "All retention measures were assigned to the facility where the care episode began." I thought this might be worth more discussion, given the high population mobility in SA - especially where you discuss the rural/urban comparison and where you discuss the possibility of people transferring care in search of better quality care. If this does occur (and it probably does to some extent) then I wonder if this might potentially introduce bias to your analysis. It would be helpful (if possible) to report in the results the proportion of people that had lab results from more than one facility (suggesting move/transfer)*

We believe our approach of attributing care episodes to the clinic where the episode began is the safest and least biased approach. The alternative – to assign the episode to the clinic where the episode ended – would imply that facilities that start patients and then refer them elsewhere (e.g. hospitals) would have downward biased quality of care. We now write in the Methods section:

“In cases of transfer, care episodes were assigned to the facility where the episode began, i.e. the sending facility, to avoid bias in retention estimates. Because unsuccessful transfers, i.e. transfers that do not lead to retention in care, are not identified in the data and cannot be linked to a receiving facility, assigning successful transfers to receiving facilities would have led to upward bias in retention at those facilities and downward bias at sending facilities.”

*4.      Methods, p6 (Viral suppression among patients monitored) - One of the limitations of having only lab data decoupled from clinical data is you can't identify those who are having VL checked when returning to care after LTF. So the LTF could be considered a metric of poor quality but then returning to care (where VL, if checked, will naturally be high) could be considered a metric of good quality care. You have a similar problem with the CD4 recovery metric. Again I recognize you can't do anything about this but just flagging the cycling in and out of care as a substantial limitation that makes inferences about quality from these data quite complicated, and might help to partly explain the low correlation of individual facility-year-level quality measures*

Great point, and we have clarified this limitation in our discussion section: “Because we do not observe more frequent clinic visits or pharmacy pick-ups, we may also miss patterns where patients cycle in and out of care between laboratory results.”

5*.      Methods, p6 (CD4 recovery) - "Some facilities have been slow to implement viral load monitoring and have relied instead on CD4 monitoring as an alternate measure of treatment outcomes." I'm not sure I agree with this statement - yes there have been problems with poor coverage and quality of routine VL monitoring but I don't think that's because people have chosen to use CD4 monitoring instead*

National guidelines specified CD4 monitoring at presentation, at treatment start, six months, 12 months, and annually thereafter until 2013, when CD4 counts were no longer collected after one year on ART. While VL monitoring was being scaled up, many facilities had already been collecting CD4 counts for years.

*6.      Table 3 - typo "District or CHC hospital" should be "District hospital or CHC", although should really spell out community health centre either in table or as footnote*

Thank you, we have made this correction.

*7.      In the discussion of the limitations of this analysis, I would prefer to see more explicit discussion of why lab data uncoupled from clinical data are imperfect for estimating quality of HIV services. What is it in particular that you miss without linked clinical data and how might this affect these inferences about quality? See comment above about retention and the challenge of people cycling in and out of care for example*

We agree and have added this to the Discussion section. We have also added: “Further linkages of laboratory data with clinical and pharmacy data could yield further quality indicators—such as visit and medication pick-up adherence—and could improve the accuracy and precision of the overall quality-of-care score.”
